# Supplementary material for: Association between oral microbiome and seven types of cancers in East Asian population: a two-sample Mendelian randomization analysis
Source: Front Mol Biosci. 2023 Nov 21;10:1327893. doi: 10.3389/fmolb.2023.1327893 (PMC10702768; doi:10.3389/fmolb.2023.1327893)
Supplement: Supplementary file 1 [file DataSheet1.ZIP › Supplementary Materials/MR plots for tongue/tongue═╝/Pancreatic cancer/╥╚╧┘░⌐_╔╕╤í╜ß╣√/pheno.2064_to_pancreatic cancer_leave_one_out.pdf]

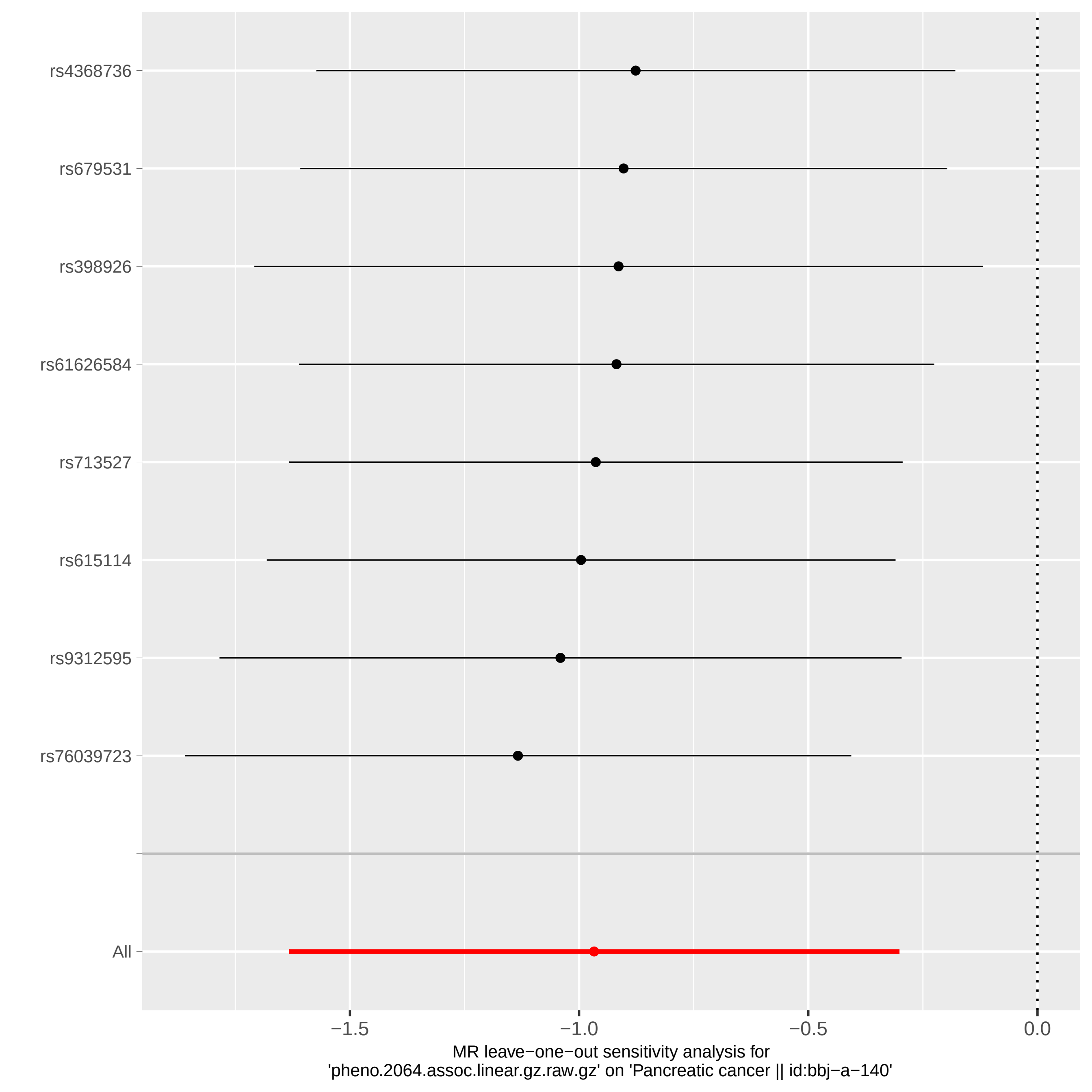
rs4368736
rs679531
rs398926
rs61626584
rs713527
rs615114
rs9312595
rs76039723
All
−1.5
−1.0
−0.5
0.0
MR leave−one−out sensitivity analysis for
'pheno.2064.assoc.linear.gz.raw.gz' on 'Pancreatic cancer || id:bbj−a−140'
